# Drunken Doctors

**Published:** 1876-08

**Authors:** 


### *DRUNKEN DOCTORS.*

Lives there a man whom some *considerate* acquaintance has not bored with stories of miracles performed by the drunken doctor of his native village? If in addition to his being a sot he happened to be an Indian or a lunatic, language is too feeble to convey an idea of his skill, and our historian only clasps his hands and mutely rolls his eyes upward. A few days ago, a Dutchman related to us his experience with a wonderful doctor at Newark. Said Hans, "I was shuse so seek as dead, ven I sees dat doctor. De doctor so trunk he can't stood up; but he shruck me un plo on mine side mit his feast, and say you got big pain dare; your *melt* ish so pig like *doo* melt, und you will die, but I can cure you. You shuse give me dwenty dollar, vich I do, und right away he make me petter as coot. I dinks he is de greatest doctor ebber lif; bot visky makes him got ded now." The Dutchman was not ashamed "to speak his mind," but many an *intelligent* American would have affected—in public—to despise the drunken quack, and would have secretly gone and consulted him. It is remarkable that education and skill are requisite to success in all other kinds of business; but the lives of human beings are intrusted to the hands of ignorant men, whose reputations rest on their brazen assurance. No wonder that an eminent New York physician said, when asked what should be done with young men who are growing up in ignorance and dissipation, "Make doctors of them."

